# *En Route* to Completion: What Is An Ideal Reference Genome?

**DOI:** 10.1016/j.gpb.2021.09.001

**Published:** 2021-09-09

**Authors:** Weihua Pan, Jue Ruan

**Affiliations:** Shenzhen Branch, Guangdong Laboratory of Lingnan Modern Agriculture, Genome Analysis Laboratory of the Ministry of Agriculture and Rural Affairs, Agricultural Genomics Institute at Shenzhen, Chinese Academy of Agricultural Sciences, Shenzhen 518120, China

Since the Human Genome Project assembled the first draft human genome 20 years ago [1], immense manpower, material, and financial resources have been cast for generating accurate, continuous, complete, and informative reference genome for each important species. Nowadays, although the qualities of these genome assemblies have been significantly improved compared to their initial draft versions, the efforts for pursuing better ones have never stopped. With the aid of the cutting-edge sequencing technologies in recent years, researchers focusing on *de novo* genome assembly have been working hard trying to address some core problems that were believed exceptionally difficult or even “unsolvable” before and to improve the reference genomes in various ways. First of all, new contig assemblers have been developed to take advantage of Pacific Biosciences (PacBio) high-fidelity (HiFi) long reads to differentiate homologous genomic sequences with only small differences and avoid sequence collapses [2], [3]. Second, Oxford Nanopore Technologies (ONT) ultra-long (UL) reads have been applied to span long dispersed repeat units and assemble long tandem repeats like centromeres [4]. Third, Hi-C data are used to generate chromosome-level phased reference genomes for diploid and polyploid species [5]. Fourth, besides single genomes, HiFi reads have been used to improve the quality of reference metagenome [6]. Fifth, in addition to linear reference genome from one individual assembly, more informative graph-based reference genomes from a group of assemblies (pan-genome) have been built to reflect the diversity and variety in population [7], [8], [9].

Among all these efforts, the ongoing “Telomere-to-Telomere” (T2T) project, which aims at building totally complete and accurate chromosome-level reference genomes of important species, is one of the most noticeable with no doubt. For a long time, due to the high difficulty in assembling tandem repeats and dispersed duplications such as centromeres, telomeres, rDNA arrays, simple sequence repeats (SSRs), retrotransposons, DNA transposons, and segmental duplications, almost all assemblies of large eukaryotic genomes (hundreds of millions to billions of base pairs) contain a large number of gaps and misassemblies. Even in the widely used human reference genome (Hg38.p13), which is considered to be of the highest-quality among all the species, there are about 151-Mb missing genomic regions, not to mention the assemblies of other animals and plants. Due to their importance in fundamental cellular processes, the absence of these repetitive regions impedes the studies of the related diseases such as cancer and infertility [10], [11], [12], limits the association and functional analyses in genetics and genomics [13], [14], and also hinders addressing open problems like centromere evolution [15]. In addition, when applied in data analyses, the incomplete reference genomes may cause unintended consequences, *e.g.*, the contamination of bacterial gene datasets by host sequences [16] and the paralogous sequence variants called as allelic variants [17]. Therefore, it is in urgent need to solve the problem of repeat assembly, and generate T2T reference genomes for humans, animals, plants, and other important species.

Due to their advantages in accuracy and length respectively, HiFi reads and UL reads provide us with an opportunity to solve this long-standing problem. By combining these two types of data, after publishing the complete human chromosome X [18] and chromosome 8 [19] as the first T2T sex chromosome and autosome, respectively, the international T2T Consortium finally successfully assembled the whole human genome in a T2T way, and opened a new T2T era in genomics [20]. This new human genome assembly was built mostly manually and is almost totally complete and correct except the rDNA regions that contain some “fake” model sequences. Practically, except human genome, it is not feasible for so many experienced scientists in genome assembly area to gather in any other assembly projects and work manually. Therefore, methodological innovations are needed for assembling T2T reference genomes more automatically. Moreover, due to the large difference between the genomes from different kingdoms (*e.g.*, animals and plants), more T2T assemblies need to be built for systematically studying the composition and structures of centromeres, rDNA arrays, and other repetitive regions.

As the most important model plant, *Arabidopsis thaliana* is one of the earliest species with draft reference genomes published [21]. However, until recently, centromeres, telomeres, and nucleolar organizing regions (NORs) have been either lost or misassembled in the existing “gold standard” reference genome. To solve these problems, Wang et al. [22] took advantage of UL, HiFi, and Hi-C data to generate a very high-quality reference genome of *A. thaliana* Col-0, named as Col-XJTU, which is almost complete with only two gaps remaining. Compared to the state-of-the-art reference genome (TAIR10.1), Col-XJTU fills 36 gaps, introduces 14.6-Mb new sequences, and improves the total size of *A. thaliana* genome to 133,725,193 bp. The new assembly contains three complete centromeres and eight complete telomeres with uniform read coverage and sizes consistent with physical map-based estimation and reported lengths. In terms of correctness, Col-XJTU improves the quality values (QVs) of all five chromosomes from QV45–52 to QV62–68. And the synteny plot shows that Col-XJTU genome is highly concordant with TAIR10.1. In terms of contiguity, the contig N50 is increased from 11.19 Mb in TAIR10.1 to 22.25 Mb in Col-XJTU. After assembling Col-XJTU, Wang et al. masked repeat elements and annotated the newly-introduced sequences. Among the 165 newly-annotated protein-coding genes, 130 are located in NORs and 35 are from centromeres and telomeres. More interestingly, they find that 96% of the newly-annotated genes are actively transcribed across different tissues, and some highly expressed leaf-specific new genes code for protein domains such as ATP synthase subunit C and NADH dehydrogenase, suggesting that they might be involved in photosynthesis.

Upon the near-T2T assembly of *A. thaliana*, Wang et al. studied the architecture, composition, and epigenetic regulation of centromeres. The *A. thaliana* genome contains a group of long centromeres ranging from 3.6 Mb to 9 Mb, which provides a chance for systematic studies of plant centromeres in a broader sense. Wang et al. discover that *A. thaliana* centromeres are composed of monomers with each around 178 bp in length (CEN180), which is different from human centromeres containing higher order repeat (HOR) units of more than 2000 bp. In addition to CEN180 repeats, GC-rich 5S rDNA sequences with hypermethylation patterns are also found in centromere regions. The majority of centromere satellite sequences show high (> 90%) inter- and intra-chromosomal identities, which is also different from human centromeres whose inter-chromosomal sequence identities are significantly lower than intra-chromosomal ones. In addition to the findings on sequence composition, they have also observed that the centromere-specific histone H3-like protein (CENH3) is significantly enriched in the interior of the centromere but depleted at the long terminal repeat (LTR) regions, and the CENH3-binding signal exhibits stronger preference for some repeat sequence clusters over the others. Moreover, five centromeres all show much higher DNA methylation than the pericentromeric regions.

In terms of methodology, Wang et al. used a novel pipeline including UL read-based assembly and HiFi read-based polish to build this near-T2T reference genome of *A. thaliana*, rather than following the technical route in human T2T assembly, which includes HiFi read-based contig assembly and UL read-based scaffolding-like process. Theoretically, the contig assemblies with HiFi reads and UL reads both have advantages in avoiding sequence collapses and generating complete and correct contigs. The high accuracy of HiFi reads is better for differentiating the repetitive units with high similarity, while the long UL reads are easier to span shorter tandem repeats and dispersed duplication units. Although the human T2T project succeeds in generating very high-quality assembly manually, it is still not clear whether its technical route is suitable for automated T2T assembly. Also, it is not clear whether the existing contig assemblers designed for normal noisy long reads are able to take fully advantage of UL reads. Therefore, until now, there is no widely-recognized “best” pipeline for T2T assembly, and more algorithmic innovation and computational experiments need to be done in this new area.

To conclude, Wang et al. built the first near-T2T reference genome of *A. thaliana*, which remarkably improves the completeness and correctness of the state-of-the-art genome. This high-quality reference genome provides a good opportunity for plant biologists to systematically study the architecture and composition of plant centromeres, telomeres, rDNA arrays, and other highly repetitive sequences, and to perform the comparative analyses with those in animals. In addition, the new UL read-based assembly pipeline proposed and implemented by Wang et al. offers a choice for generating T2T reference genome in a more automated way.

## CRediT author statement

**Weihua Pan:** Writing - original draft. **Jue Ruan:** Conceptualization, Writing - review & editing. Both authors have read and approved the final manuscript.

## Competing interests

The authors have declared no competing interests.
